# Antiseizure medications and oral contraceptives: Impact of enzyme inducers on pregnancy outcomes and costs

**DOI:** 10.1016/j.yebeh.2021.108368

**Published:** 2021-12

**Authors:** Seri Anderson, Josephine Mauskopf, Sandra E. Talbird, Annesha White, Meenakshi Srinivasan

**Affiliations:** a RTI Health Solutions, 3040 Cornwallis Road, Research Triangle Park, NC 27709, USA; b University of North Texas System College of Pharmacy, 3500 Camp Bowie Blvd, IREB Office 211, Fort Worth, TX 76107, USA

**Keywords:** Oral contraceptives, Antiseizure medications, Drug–drug interactions, Unintended pregnancy, Costs

## Abstract

**Purpose:**

To show the impact of drug–drug interactions (DDIs) associated with co-administration of enzyme-inducing (EI) antiseizure medications and oral contraceptives (OCs) on the annual number of unintended pregnancies, their outcomes, and their associated costs in the United States (US).

**Methods:**

A Microsoft Excel pregnancy-outcomes model was developed to determine the impact of DDIs in women who take an OC as well as an EI antiseizure medication known to lower the effectiveness of the OC in preventing pregnancy. The model compared the number of unintended pregnancies, the expected pregnancy outcomes, and associated costs in women taking an OC and an EI medication with a matched cohort of women who took an OC and an enzyme-neutral (EN) antiseizure medication that is known not to interact with OCs. The model perspectives were patients and third-party payers in the US. Unintended pregnancy rates, pregnancy outcomes, and cost inputs for the model were taken from published studies.

**Results:**

The results of the analysis showed an estimated increase in the annual number of unintended pregnancies in the US of 503 (a change from 1151 to 1654), an increase of 44.7%, for the estimated 71,922 women currently taking an OC plus an EI medication in the US when compared with a matched cohort taking an OC plus an EN medication. This resulted in an estimated annual healthcare cost increase of $3 million, which is an increase of 5.5% in the annual costs for contraception and pregnancy care. A scenario analysis showed that the annual number of unintended pregnancies could be lower (575 vs 1654) for a matched cohort of women taking EI medications and using a copper intrauterine device, a highly effective and nonhormonal form of contraception, rather than an OC.

**Conclusions:**

Physicians treating women of reproductive age for epilepsy who wish to avoid pregnancy should consider the potential for DDIs that might result in unintended pregnancies. Thus, physicians should alert women using EI medications for epilepsy control to the increased potential for unintended pregnancies if they use OCs for contraception.

## Highlights

• Enzyme inducing antiseizure medications reduce oral contraceptive efficacy.
• This drug–drug interaction may cause >500 unintended pregnancies annually in the US.
• Unintended pregnancies may harm the health of women with epilepsy and their infants.
• This drug interaction may increase annual pregnancy healthcare costs by $3 million.
• Readers can use the online Excel model to calculate these outcomes for other regions.

## 1 Introduction^2^

Women with epilepsy report that 50% of their pregnancies are unplanned [1]. For some women, unintended pregnancies may be mistimed but not unwanted and may not lead to negative outcomes; however, recent studies comparing women who received abortions with those who were denied abortions found that unintended pregnancies, if carried to term, were associated with worsened health and long-term economic hardship for the woman, in addition to exposure to the life-threatening risks of childbirth [2]. For women with epilepsy, pregnancies can also increase seizure frequency [3]. However, planned pregnancies rather than unintended pregnancies are associated with better seizure control and less fetal exposure to antiseizure medications, which can cause pregnancy complications, congenital malformations, and other poor birth outcomes [4].

Unintended pregnancies mainly result from contraceptive nonuse or incorrect or inconsistent use of effective contraceptives [5]. The most commonly used effective and reversible contraceptive method among women in the United States (US) [6] and among women with epilepsy [1] is oral contraceptives (OCs). Although unintended pregnancies can occur among women who are fully adherent to their OCs [7], [8], [9], contraceptive failures also occur due to drug–drug interactions (DDIs) between OCs and antiseizure medications [10].

The potential for DDIs between OCs and antiseizure medications has been known for many years. In 1972, a letter published in the *British Medical Journal* [11] documented a pregnancy in a woman who was fully compliant with an OC regimen while also taking drugs to control her epilepsy. More recently, a study of 1144 women in the Epilepsy Birth Control Registry examined the risk of unintended pregnancies and the antiseizure medication and type of contraceptive used at conception in the 78.9% of women who reported at least one unintended pregnancy [12]. The authors found that those women using systemic hormonal contraception combined with an enzyme-inducing (EI) antiseizure medication (such as phenytoin, carbamazepine, topiramate, phenobarbital, or oxcarbazepine) had a substantially greater rate of unintended pregnancies than those using other combinations of contraception and enzyme-neutral (EN) antiseizure medication (such as lamotrigine, valproate, gabapentin, or levetiracetam).

A recent commercial claims database analysis has estimated the contraceptive failure rates among users of concomitant EI and EN antiseizure medications [13]. This study showed an increased rate of contraceptive failure among women with epilepsy or bipolar disorder taking concomitant EI antiseizure medications. The consequences of these DDIs, which may result in substantial distress for women experiencing the unintended pregnancy, are addressed in a forthcoming US cost-effectiveness analysis studying women with chronic comorbid conditions requiring the use of medications that interact with OCs, including antiseizure medications [unpublished, White, A; Lott, J; Williamson, T; Kong, S; Plouffe, L. Quantifying the economic burden of unintended pregnancies due to drug-drug interactions with hormonal contraceptives from the United States payer perspective]. The current study estimated, from the perspective of women of reproductive age with epilepsy in the US, the annual number of unintended pregnancies due to DDIs of EI antiseizure medications with OCs and their outcomes—live birth, ectopic pregnancy, spontaneous abortion, or abortion. From the perspective of US payers, the analysis also estimated the healthcare costs associated with these outcomes. An interactive model is included in the Supplementary Material and can be readily adapted to estimate the number of unintended pregnancies, their outcomes and associated healthcare costs in other regions.

## 2 Material and methods

A Microsoft Excel pregnancy-outcomes model was developed to determine the impact of DDIs in women of reproductive age with epilepsy who take an OC plus an antiseizure medication that is known to interact with OCs by lowering the effectiveness of the OC in preventing pregnancy (hereafter referred to as EI antiseizure medications). The model compared the number of unintended pregnancies and their outcomes (including live births and abortions) and pregnancy-related costs in these women with a matched cohort of women who took an OC and an antiseizure medication that is known not to interact with OCs (hereafter referred to as EN antiseizure medications). Previous key clinical and cost-effectiveness analyses of antiseizure medication have not included the outcomes and healthcare costs associated with DDI-related unintended pregnancies [14], [15]. The model took the perspectives of women with epilepsy and payers in the US. The perspective of women with epilepsy includes all health outcomes, while the payer perspective includes all pregnancy-related costs. The model was designed to be able to be used in different regions or countries using local inputs for the covered population characteristics, EI antiseizure medication usage rates, and outcomes and costs of unintended pregnancies.

### 2.1 Model structure

The modeled population consisted of US women of reproductive age (18–44 years) with epilepsy who did not wish to become pregnant and who were sexually active. The model estimated the number of women taking OCs in the US who also took an EI antiseizure medication (OC + EI cohort) and compared them to an age-matched group of women taking OCs in the US who also took EN antiseizure medication (OC + EN cohort). Women in the OC + EI cohort were at increased risk of unintended pregnancy due to DDI, and women in the OC + EN cohort were not. The economic analysis was conducted over a 1-year time horizon. The primary outcomes of interest were the differences in annual DDI-related unintended pregnancies, pregnancy outcomes, and payer pregnancy-related costs reported for the two cohorts.

**Fig. 1:**
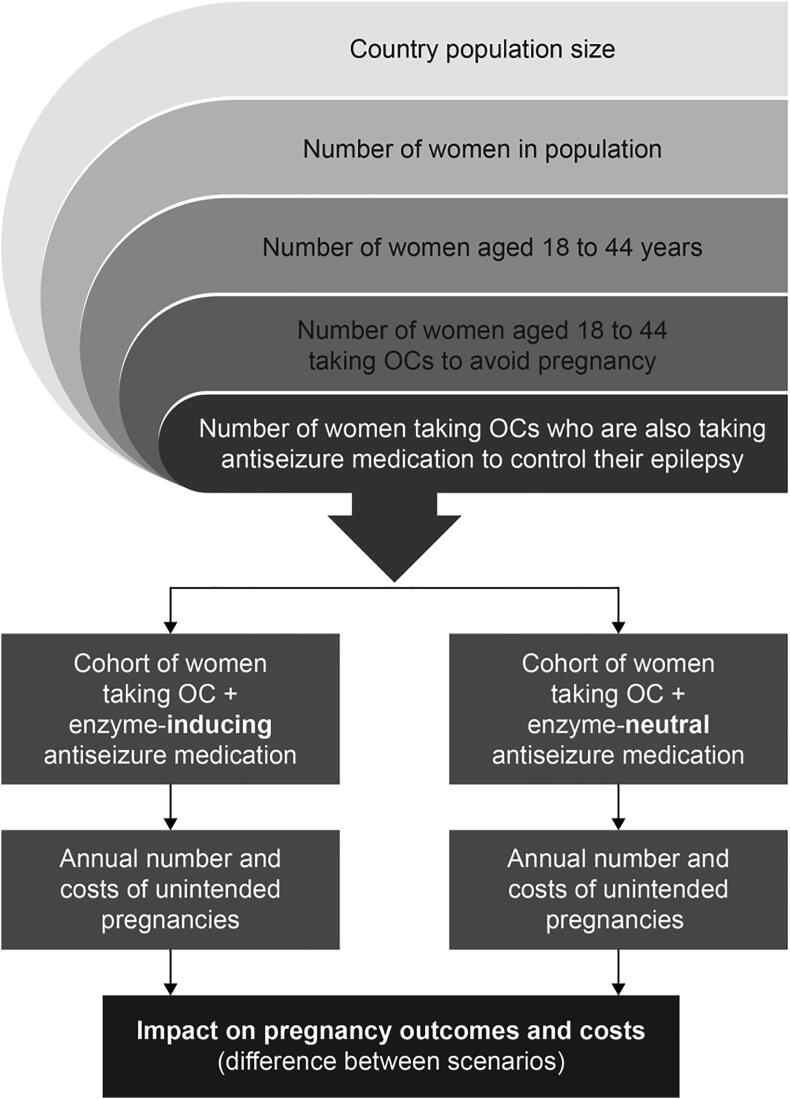
Pregnancy-outcomes model structure. Note: The size of the population at risk of DDIs due to OCs and enzyme-inducing antiseizure medications is estimated. Then, two cohorts are compared, one taking OC + enzyme-inducing medications and one taking OC + enzyme-neutral medications. The annual number and outcomes and costs of unintended pregnancies for each cohort are calculated, and the difference in these outcomes between the two cohorts is the DDI impact. DDI = drug–drug interaction; OC = oral contraceptive.

Population-level costs and outcomes for the US were estimated according to the age distribution of women with epilepsy in the two population cohorts of interest. Age matching was used because fertility rates and contraceptive use may vary by age. Fig. 1 presents a general overview of the modeling approach.

The model was built with default input values and references in the base-case analysis and with flexible input values in the user-defined cells so that a user can easily modify key input parameters to adapt the model to other regions or countries of interest (see Supplementary Materials). The model used inputs from the published literature and publicly available data sets only and as such IRB approval for the research was not required.

### 2.2 Model inputs

To estimate the outcomes and costs, the following input parameters were included in the model:

• Population characteristics
• Unintended pregnancy rates and costs
• OC costs

We estimated the number and age distribution of women of reproductive age in the US who were taking OCs as well as EI or EN antiseizure medications, using data from the Centers for Disease Control and Prevention [16] and the published literature. The number of women taking an OC and an EI medication was then estimated using data from a recent study (see Table 1). An age-matched population of equal size was created for women taking OCs and an EN medication. Table 1 presents the estimated size of the two population cohorts. Table 2 presents the number of women in the model by age group.

**Table 1:** Population data on the number of women and percentage of women using OCs and antiseizure medications in the US.

Variable	Value	Reference
Total population of US, *n*	328,239,523	Estimate for 2019 [17]

Total female population, *n*	166,582,199	Estimate for 2019 [17]

Total female population of reproductive age, *n* (%)	64,325,356	Estimate for 2019 [17]
15–19 y	10,308,963 (16.0)	Estimate for 2019 [17]
20–24 y	10,568,188 (16.4)	
25–29 y	11,504,446 (17.9)	
30–34 y	11,076,695 (17.2)	
35–39 y	10,852,580 (16.9)	
40–44 y	10,014,484 (15.6)	

Women using OCs, %		Daniels and Abma [6]
15–19 y^a^	19.5	
20–24 y	21.6	
25–29 y	21.6	
30–34 y	10.9	
35–39 y	10.9	
40–44 y	6.5	

Women aged 18–44 y who used an antiseizure medication in the past 30 d, %^b^	4.2	Estimate for 2011–2014 [16]

Exposure among 108,741 OC + antiseizure medication users, %^c^		Sarayani et al. [13]
Enzyme-inducing medication	17.4	
Enzyme-neutral medication	82.6	

CDC = Centers for Disease Control and Prevention; OC = oral contraceptive; US = United States.

a Value is based on the 15- to 19-year-old age group, but was applied in the model to the 18- to 19-year-old women.

b Antiseizure medications include the following classes in the CDC definition: hydantoin, succinimide, barbiturate, benzodiazepine, miscellaneous, dibenzoazepine, fatty acid derivative, gamma-aminobutyric acid reuptake inhibitors, gamma-aminobutyric acid analogs, triazine, carbamate, pyrrolidine, and carbonic anhydrase inhibitor.

c Study considered carbamazepine (enzyme inducing), oxcarbazepine (enzyme inducing), lamotrigine (enzyme neutral), or levetiracetam (enzyme neutral).

**Table 2:** Women included in the model.

Age Group, y	Data Label for Calculations						
	A	B	C	D	E	F	G
	Women of Reproductive Age in the US, n	Women Using Antiseizure Medication, %	Women Using Antiseizure Medication, n	Women Using OCs, %	Women Using OCs and an Antiseizure Medication, n	Women Using an EI Medication, %	Women Using OCs and an EI Medication, n
15–19	10,308,963	4.2	432,976	19.5	84,430	17.4	14,724
20–24	10,568,188	4.2	443,864	21.6	95,875	17.4	16,720
25–29	11,504,446	4.2	483,187	21.6	104,368	17.4	18,201
30–34	11,076,695	4.2	465,221	10.9	50,709	17.4	8,843
35–39	10,852,580	4.2	455,808	10.9	49,683	17.4	8,665
40–44	10,014,484	4.2	420,608	6.5	27,340	17.4	4,768
**Total**	**64,325,356**		**2,701,664**		**412,405**		**71,922**
Source	Estimate for 2019 [17]	Estimate for 2011–2014 [16]	Calculation (A * B)	Daniels and Abma [6]	Calculation (C * D)	Sarayani et al. [13]	Calculation (E * F)

EI = enzyme-inducing; CDC = Centers for Disease Control and Prevention; OC = oral contraceptive; US = United States.

Incidence of unintended pregnancies was estimated from the published literature [13] on unintended pregnancy rates in women taking OC + EI antiseizure medications or OC + EN antiseizure medications. The Pearl Index was used to derive from the literature the annual probability of contraceptive failure [5], [18], [19]. Sarayani and colleagues [13] used US commercial claims data from IBM MarketScan® from 2005 to 2017 to estimate unintended pregnancy rates among 108,741 users of either EN or EI antiseizure medications (specifically carbamazepine [EI], oxcarbazepine [EI], lamotrigine [EN], or levetiracetam [EN]) during episodes in which an OC was concomitantly taken. Based on the data analyzed by Sarayani et al. [13], we assumed that EN and EI antiseizure medications were not taken simultaneously and that the antiseizure medications and OCs were taken concomitantly, which means that the potential for interaction of the drugs would be at its highest. We also assumed that antiseizure medications were selected to optimize seizure control and minimize other adverse effects in both the EI and EN antiseizure medication groups.

Outcomes associated with unintended pregnancies are ectopic pregnancy, spontaneous abortion, induced abortion, and preterm or full-term births. Input data for these outcomes are presented in Table 3. The age-dependent total numbers of pregnancies, live births, induced abortions, and spontaneous abortions/ectopic pregnancies in the US general population were taken from Ventura et al. [20]. To derive separate estimates for the number of spontaneous abortions and ectopic pregnancies, we calculated the number of ectopic pregnancies using rates from Hoover et al. [21]. The age-dependent probability that each birth outcome was due to an unintended pregnancy was based on estimates from Finer and Zolna [22], Finer and Henshaw [23], and Mosher et al. [24]. Finally, those estimates were used to calculate the age-weighted values for the pregnancy outcomes of unintended pregnancies.

**Table 3:** Annual unplanned pregnancy rates.

Input Parameter	Base Case	Lower Bound	Upper Bound	Source
**Unplanned pregnancy rates per 100 persons per year with OC**				
Unplanned pregnancy: OC + EN	1.6	1.4	1.8	Base case: Sarayani et al. [13]. Lower and upper bound: 95% CI from Sarayani et al. [13]
Unplanned pregnancy: OC + EI	2.3	1.9	2.8	

**Outcome of unplanned pregnancies, %**				
Birth	49.2	30	69	Base case: calculated from the literature [20], [21], [22], [23], [24]
Induced abortion	35.0	14	58	
Spontaneous abortion	15.3	11.5	16	Lower and upper bound: minimum and maximum abortion rates with associated pregnancy outcomes from Kost et al. [25]
Ectopic pregnancy	0.5	NA	NA	Assumption

CI = confidence interval; EI = enzyme-inducing antiseizure medication; EN = enzyme-neutral antiseizure medication; NA = not applicable; OC = oral contraceptive.

The model considered only the following direct medical costs:

• Costs associated with unintended pregnancy, including ectopic pregnancy, induced abortion, or spontaneous abortion as well as for delivery for those pregnancies ending in a live birth (but not the cost postdelivery)
• Costs associated with OCs

Lifetime costs for children born that may be covered by the payer were not included. The costs used in the model were taken from the published literature (see Table 4).

**Table 4:** Annual or per-event costs (in 2020 US Dollars).

Input Parameter	Base Case	Lower Bound	Upper Bound	Source
**OC cost per year**				
OC	$850.81	$425.41	$1276.22	Annualized OC cost estimates [26] adjusted to 2020 US dollars using the CPI medical care component [27]. Lower and upper bound +/- 50%.
**Costs associated with unintended pregnancy, per event**				
Birth	$12952.67	$5269.86	$28664.46	Base case and lower and upper bounds: literature review^a^
Spontaneous abortion	$1121.01	$600.84	$3593.84	
Induced abortion	$939.92	$600.84	$4233.11	
Ectopic pregnancy	$6174.08	$2839.92	$15943.46	

CPI = Consumer Price Index; OC = oral contraceptive; US = United States.

a A literature review was conducted for the cost of each pregnancy outcome and the median, minimum, and maximum costs across the cited articles were calculated (with all prices adjusted to 2020 US dollars) [19], [26], [28], [29], [30], [31], [32], [33], [34], [35], [36], [37], [38]. These articles were identified using a registry of economic evaluations of hormonal contraceptives [39].

### 2.3 Sensitivity and scenario analyses

The robustness of model assumptions and uncertainty around the key input parameters were tested in one-way sensitivity analyses using their estimated lower or upper bounds. The model user could enter an alternative lower or upper bound, if desired. Model settings (e.g., time horizon) and population characteristics (e.g., age distribution) were not varied in the one-way analysis.

A scenario analysis was also performed comparing women taking an OC + EI antiseizure medication with those using a copper intrauterine device (IUD) for contraception + EI antiseizure medication. The copper IUD is the only highly effective, nonhormonal method of contraception available, so it would not produce DDIs when taken with an EI antiseizure medication [40]. The copper IUD was assumed to have an unplanned pregnancy rate of only 0.8 per 100 persons per year and an annualized cost of $174.66 [26], [41]. This cost was calculated based on the device, insertion, removal, and monitoring costs ($1,362.37) that were annualized over 7.8 years of use, with the assumption that a 22% dropout rate in the first year of use would occur [28], and was adjusted to 2020 US dollars using the medical care component of the CPI [27].

### 2.4 Model assumptions

The assumptions used in the model are listed below:

• The analysis considered only women aged 15–44 years who were sexually active and who did not intend to become pregnant during the time horizon of the analysis. Therefore, all pregnancies occurring in women taking an OC during the time horizon that resulted from method failure were assumed to be unintended.
• The model used a single point estimate for the probability of each pregnancy outcome that was age-weighted to the US female population of reproductive age based on the age-specific values in Ventura et al. [20] from the US general population; upper and lower bounds for each probability were based on estimates from the literature [25].
• All women modeled were assumed to have preexisting epilepsy that necessitated taking antiseizure medication.
• We assumed that all OCs had the same propensity for a DDI with an EI antiseizure medication and that all women modeled were taking low-dose estrogen (<50 μg) or progestin-only OCs.
• We assumed that women did not switch between contraceptive methods during the time horizon of the study.
• We assumed that women used OC for a full year unless they experienced an unintended pregnancy, in which case they discontinued their OC.
• Contraceptive failure occurred at the midpoint of the model year. However, costs associated with births, induced abortions, spontaneous abortions, and ectopic pregnancies were assumed to take place in the same year as the unplanned pregnancy.
• Long-term costs from live births were not included in the model.
• We assumed that a woman could get pregnant only once per year.

## 3 Results

### 3.1 Base-case analysis

The model calculated the pregnancy and cost outcomes for a 1-year time horizon. Annual pregnancy outcomes, annual costs of pregnancy outcomes and OCs, and the total costs per woman at risk of DDIs (*n* = 71,922) are shown in Table 5 for each cohort, along with the difference (DDI impact) between the two cohorts.

**Table 5:** Annual pregnancy outcomes and costs for comparator populations and DDI impact.

Parameter	OC + EI	OC + EN	DDI Impact:
	(*N* = 71,922)	(*N* = 71,922)	OC + EI Minus OC + EN
**Pregnancy outcomes**			
Number of unintended pregnancies	1654	1151	503
Number of unintended live births	814	566	248
Number of ectopic pregnancies	8	6	2
Number of spontaneous abortions	253	176	77
Number of induced abortions	579	403	176
**Total annual costs**	$71,205,253	$68,157,724	$3,047,530
Cost of unintended pregnancy outcomes	$11,420,726	$7,944,853	$3,475,873
Cost of OCs	$59,784,527	$60,212,871	−$428,344
**Total annual costs/Number of women at risk (N)**	$990	$948	$42

DDI = drug–drug interaction; EI = enzyme-inducing antiseizure medication; EN = enzyme-neutral antiseizure medication; OC = oral contraceptive.

Drug–drug interactions associated with the use of an OC + EI antiseizure medications resulted in 503 additional unintended pregnancies, resulting in 248 unintended births and 256 pregnancies ending in ectopic pregnancy, spontaneous abortion, or induced abortion. This resulted in an estimated increase in healthcare costs of a little over $3 million from the US payer perspective annually among women of reproductive age with epilepsy.

### 3.2 One-way sensitivity analyses and scenario analysis

Table 6 presents the results of the one-way sensitivity analyses.

**Table 6:** Results of one-way sensitivity analyses.

Parameter	DDI Impact: OC + EI Minus OC + EN		
	Incremental Costs	No. of Additional Unintended Pregnancies Due to DDIs	No. of Additional Unintended Live Births Due to DDIs
Base case^a^	$3,047,530	503	248
Low failure rates for contraception (OC + EI = 1.9, OC + EN = 1.4)	$2,176,807	360	177
High failure rates for contraception (OC + EI = 2.8, OC + EN = 1.8)	$4,353,614	719	354
Low abortion rate	$4,275,882	503	350
(induced abortion = 14%, birth = 69.5%, spontaneous abortion = 16%, ectopic = 0.5%)			
High abortion rate	$1,882,878	503	151
(induced abortion = 58%, birth = 30%, spontaneous abortion = 11.5%, ectopic = 0.5%)			
Low annual contraceptive costs	$3,261,701	503	248
(OC = $425.41)			
High annual contraceptive costs	$2,833,358	503	248
(OC = $1276.22)			
Low unintended pregnancy costs	$1,036,298	503	248
(birth = $5269.86, induced abortion = $600.84, spontaneous abortion = $600.84, and ectopic pregnancy = $2839.92)			
High unintended pregnancy costs	$7,734,678	503	248
(birth = $28664.46, induced abortion = $4233.11, spontaneous abortion = $3593.84, ectopic pregnancy = $15943.46)			

DDI = drug–drug interaction; EI = enzyme-inducing antiseizure medication; EN = enzyme-neutral antiseizure medication; OC = oral contraceptive.

a Base-case values for contraceptive failure rates are 2.3 for OC + EI and 1.6 for OC + EN. Base-case outcomes for unintended pregnancies were 35% for induced abortion; 49.2%, live birth; 15.3%, spontaneous abortion; and 0.5%, ectopic pregnancy. Base-case annual costs for OC are $850.81. Base-case costs for pregnancy outcomes were $12,953 for live birth; $940, induced abortion; $1121, spontaneous abortion; and $6174, ectopic pregnancy.

The results were robust to changes in the key parameters; women taking an EI antiseizure medication had an increased number of unintended pregnancies and increased costs in all one-way sensitivity analyses when compared with women taking an EN antiseizure medication and an OC.

An additional scenario analysis was performed comparing women using an OC and an EI antiseizure medication with women using a copper IUD for contraception and an EI antiseizure medication. This scenario found that there were almost three times as many unintended pregnancies in the OC + EI antiseizure medications group as compared with the IUD + EI antiseizure medications group (1654 vs. 575 = 1079 additional unintended pregnancies) because of the greater effectiveness of IUDs versus OCs and the lack of an interaction with the EI medication. This scenario also found a lower total cost per woman at risk of a DDI for women using a copper IUD (annual contraceptive costs of $228 per woman for the IUD + EI antiseizure medications cohort vs. $990 per woman at risk of a DDI for the OC + EI antiseizure medications cohort).

## 4 Discussion

The pregnancy-outcomes model estimated the number of unintended pregnancies and the associated costs because of DDIs for women with epilepsy who are taking an OC combined with an EI antiseizure medication rather than combined with an EN antiseizure medication. Similar estimates of the risk of unintended pregnancies for women taking an OC and an EI antiseizure medication have been shown in a registry study [12] and in an observational database study [13]. The clinical effectiveness and cost-effectiveness in terms of seizure control of various antiseizure medications have been previously studied in the SANAD [14] and SANAD II [15] trials. These trials found that lamotrigine (EN) and valproate (EN) were preferred to carbamazepine (EI), topiramate (EI), gabapentin (EN), oxcarbazepine (EI), and levetiracetam (EN) depending on the population studied [40]. However, valproate is not recommended for women at risk of pregnancy due to its teratogenicity, and the study authors acknowledged that weighing the costs and benefits of improved seizure control versus reduced teratogenicity could only be done by individual women [15]. Our study has added to these findings by estimating the impact of different EI or EN antiseizure medications on unintended pregnancies and their outcomes and associated healthcare costs in women using an OC for contraception and experiencing a DDI-related unintended pregnancy in the US. In addition, the pregnancy-outcomes model included in the Supplementary Material can easily be used to create estimates for other regions and countries.

The model has several limitations. First, the short time horizon did not reflect that contraception and antiseizure medications are typically taken for many years. Women may change their behavior after an unintended pregnancy due to a DDI-related OC failure. However, the short time horizon mitigated the issues of tracking trajectories of contraceptive use over time and modeling the phenomenon of repeat unintended pregnancies. Second, the model used estimates from the general population to estimate the number of women aged 15–44 with epilepsy at risk of a DDI. To address this concern, we attempted to validate our estimate of the population size of women receiving both an antiseizure medication and an OC (*n* = 71,922) using estimates from the epilepsy-specific literature. Using an alternative approach, a similar estimate of the size of the population at risk was derived. Third, the estimate of OC failure rates with EI and EN medications used in this article was developed by Sarayani et al. [13] in a study of women taking one of four medications: carbamazepine and oxcarbazepine for EI medications and lamotrigine and levetiracetam for EN medications. More research is needed on the impact of DDIs on hormonal contraceptive failure rates in women taking antiseizure medication, including those taking multiple medications. However, in the sensitivity analyses, we show how the number of unintended pregnancies, pregnancy outcomes, and costs changed when DDIs between OC and other EI medications resulted in smaller or larger OC failure rates than the failure rates used in the base case. In addition, we assumed that EN and EI medications are not taken simultaneously, which may not be the case for patients on polytherapy. However, treatment guidelines recommend polytherapy only when multiple monotherapy trials have failed, and these guidelines appear to be widely followed in the US, suggesting that many women with epilepsy match these assumptions [42]. Finally, the cost projections are estimated using published data on unintended pregnancies for all US women taking OCs.

Registry and observational findings [12], [13] suggest that DDIs may not routinely be considered when making contraceptive decisions for women with epilepsy. Several reasons might explain why this happens, including different physicians prescribing for the different conditions, a lack of readily available information on possible DDIs, or the complexity of DDIs to consider when co-prescribing contraception and antiseizure medications. Electronic medical records that ensure prescribers know of other medications, including but not restricted to contraception being taken by their female patients with epilepsy as well as pop-up warning notices that warn them about DDIs of newly prescribed medical combinations, can address this issue for women with epilepsy taking antiseizure medications.

## 5 Conclusions

The results of the analysis showed that women currently taking an OC plus an EI medication could reduce their risk of unintended pregnancy attributable to DDIs either by substitution of an alternative form of effective, nonhormonal contraception, such as a copper IUD in place of hormonal-based OC [10], or by use of an EN medication, if this will provide adequate control of their epilepsy. The American College of Obstetricians and Gynecologists also recommends depot-medroxyprogesterone acetate injections (commonly known by the brand name Depo Provera) and the hormonal IUD as highly effective contraception options that do not demonstrate the same DDIs as OC with EI antiseizure medications [43]. Our analysis also showed that changes to reduce the risk of unintended pregnancies associated with DDIs between an OC and an EI medication could reduce costs for all US payers by $1,036,298 to $55,655,035 annually and avert between 360 and 1079 unintended pregnancies annually among reproductive-aged women with epilepsy. While this is only a small portion of the unintended pregnancies in the general population, this is a heavy burden for women with epilepsy, who may experience even greater negative impacts associated with unintended pregnancy than healthy women due to the possibility of increased seizures and negative health and developmental impacts on any children born [4]. For these reasons, careful consideration by women and their physicians should be given to the potential for DDIs that might result in unintended pregnancies when selecting contraceptive methods and/or antiseizure medications for the treatment of chronic epilepsy.

## Declaration of Competing Interest

Seri Anderson, Josephine Mauskopf, Sandra Talbird, Annesha White, and Meenakshi Srinivasan have no conflicts of interest.

## Footnotes

2 CDC = Centers for Disease Control and Prevention, CI = confidence interval, DDI = drug-drug interaction, EI = enzyme-inducing antiseizure medication, EN = enzyme-neutral antiseizure medication, IUD = intrauterine device, NA = not applicable, OC = oral contraceptive, US = United States.
